# Comparative Analysis of *Intra*- and *Inter*-Specific Genomic Variability in the Peach Potato Aphid, *Myzus persicae*

**DOI:** 10.3390/insects10100368

**Published:** 2019-10-22

**Authors:** Mauro Mandrioli, Deborah Salvatore, Agnese Ferrari, Niccolò Patelli, Gian Carlo Manicardi

**Affiliations:** Dipartimento di Scienze della Vita, Università di Modena e Reggio Emilia, Via Campi 213/D, 41125 Modena, Italy; 224541@studenti.unimore.it (D.S.); agnese.ferrari@unimore.it (A.F.); 82225@studenti.unimore.it (N.P.); manigi04@unimore.it (G.C.M.)

**Keywords:** clone, duplication, deletion, histones, aphids

## Abstract

The availability of genomic data in the last decade relating to different aphid species has allowed the analysis of the genomic variability occurring among such species, whereas *intra*-specific variability has hitherto very largely been neglected. In order to analyse the *intra*-genomic variability in the peach potato aphid, *Myzus persicae*, comparative analyses were performed revealing several clone-specific gene duplications, together with numerous deletions/rearrangements. Our comparative approach also allowed us to evaluate the synteny existing between the two *M. persicae* clones tested and between the peach potato aphid and the pea aphid, *Acyrthosiphon pisum*. Even if part of the observed rearrangements are related to a low quality of some assembled contigs and/or to the high number of contigs present in these aphid genomes, our evidence reveals that aphid clones are genetically more different than expected. These results suggest that the choice of performing genomes sequencing combining different biotypes/populations, as revealed in the case of the soybean aphid, *Aphis glycines*, is unlikely to be very informative in aphids. Interestingly, it is possible that the holocentric nature of aphid chromosomes favours genome rearrangements that can be successively inherited transgenerationally via the aphid’s apomictic (parthenogenetic) mode of reproduction. Lastly, we evaluated the structure of the cluster of genes coding for the five histones (H1, H2A, H2B, H3 and H4) in order to better understand the quality of the two *M. persicae* genomes and thereby to improve our knowledge of this functionally important gene family.

## 1. Introduction

In the last few decades several insect genomes have been sequenced, assembled and annotated covering six different orders belonging to both holometabolous and hemimetabolous species [1,2,3,4,5,6]. Furthermore, current genomic data are not limited to highly studied ‘model’ insects such as *Drosophila melanogaster and Anopheles gambiae*, but also other species, like the honey bee *Apis mellifera* [1], the red flour beetle *Tribolium castaneum* [3], the yellow fever mosquito *Aedes aegypti* [7], the mosquito *Culex quinquefasciatus* [4], and the blood-sucking bug, *Rhodnius prolixus* [6].

In view of their relevance as serious pests of a variety of crops of agricultural and horticultural importance , several aphid species (Hemiptera: Aphididae) have been studied to date, whilst economic resources are available for the pea aphid, *Acyrthosiphon pisum* [8], the Russian wheat aphid, *Diuraphis noxia* [9], the soybean aphid, *Aphis glycines* (from a composite of multiple North American populations) [10], the cotton aphid *Aphis gossypii* [11], as well as for additional species whose genomes are available in *Aphidbase,* even if their papers are still unpublished: *i.e*. *Aphis glycines* biotype 1, the black cherry aphid, *Myzus cerasi*; the peach potato aphid, *Myzus persicae;* and the bird cherry-oat aphid, *Rhopalosiphum padi.*

Genomic resources have prompted several comparisons among species and this approach has allowed, for instance, the identification of the molecular pathways evolving in each aphid species, sometimes fast, and/or the study of gene families, the functions of which are related to environmental adaptation [11,12], together with the identification of species-specific gene expansions or losses [12]. Interestingly, even if aphid genomic resources also include genome assemblies of biotypes/clones belonging to a same species [10], aphid *intra*-specific variability has been very largely neglected, despite the usefulness of this comparison. Indeed, *intra*-specific comparative analysis could indeed be useful to validate genomic data, more especially considering the fact that aphid genomes are currently scattered in thousands of contigs, thereby making the evaluation of their quality difficult.

The need for this validation is strongly supported by recent analyses pointing to the occurrence of a very high rate of misassembly in the *A. pisum* genome [13]. Indeed, Jaquiéry et al. [13] identified widespread errors in more than half of the contigs larger than 150kb, suggesting that the pea aphid genome presents considerable assembly problems to a degree that goes beyond the assembly results obtained with current assembly procedures and protocols [14,15]. 

In view of the availability of genomic resources related to two clones of the peach potato aphid *Myzus persicae* (Sulzer) in the present study, we analysed the occurrence of *intra*- and *inter*-specific genomic changes in these clones, using the pea aphid *A. pisum* genome as a reference in order to understand the quality of the assembled *M. persicae* genomes and to identify syntenic regions between clones and among aphid species. At the same time, we analysed the structure of the cluster of genes coding for histones H1, H2A, H2B, H3 and H4 in order to better evaluate the quality of the two *M. persicae* assembled genomes as well as to improve our knowledge of this gene family.

## 2. Materials and Methods 

Genomic data related to *M. persicae* clones G006 and O were downloaded from *Aphidbase* (http://bipaa.genouest.org/sp/myzus_persicae/). The genomes of *M. persicae* clones G006 and O were derived from the sequencing of a single aphid clone, whereas the *A. glycines* genome, currently available in Genbank and referred as a “hybrid” genome, derived from the simultaneous sequencing of assembling of both laboratory colonies and natural populations collected from soybean fields [10]. The pea aphid genome was downloaded from the website *Aphidbase* (http://bipaa.genouest.org/sp/acyrthosiphon_pisum/) and has been used as a genomic reference since it is, at present, the most studied and carefully annotated aphid genome.

Dot plot comparisons (visualized as 2D scatter plots) and search for syntenic regions/genes were done using the freely available software *SynFind* (https://genomevolution.org/coge/SynFind.pl) that generates syntenic dot plots [16] and *SynMap* (https://genomevolution.org/coge/SynMap.pl) that in turn reveals syntenic regions [17]. *SynFind* analyses were performed using the BLAST-variant *last* with a gene window size of 40 and setting 4 as the minimum number of gene. The scoring function “collinear” was used so that collinear arrangement of syntenic genes was enabled. *SynMap* analysis was performed setting "relative gene order" as DAGChainer option, since the absolute distance between genes in nucleotides may vary widely between genomes and even within a genome. In view of the low synteny observed in previous analyses [18], a minimum number of 5 gene-pairs was inserted as a DAGChainer search parameter. The combined use of *SynFind* and *SynMap* allowed the identification of both orthologous genes (or regions of homology) between two genomes and collinear sets of genes (or regions) of sequence similarity to infer synteny. 

The gene order obtained in *SynMap* for each aphid genome was verified using the *JBrowse* genome browser available in *Aphidbase* (http://bipaa.genouest.org/).

DNA extraction was performed using the *Wizard^®^ SV Genomic DNA Purification System* (Promega), according to the manufacturer’s instructions using 30 female adults. DNA samples were quantified by spectrophotometric absorbance measurements at a wavelength of 260 nm using a Nanodrop ND 1000 *Spectrophotometer* (*NanoDrop* Technologies). 

The Long PCR Enzyme Mix (Fermentas) was used to amplify the complete histone gene cluster using oligonucleotide primers (Table 1) specifically designed on *M. persicae* sequences with the freely available on-line tool *Primer 3* (http://bioinfo.ut.ee/primer3/) [19].

DNA sequencing was performed with Sanger sequencers at BMR Genomics (Padua, Italy), whereas the search for matrix recognition sites was done using the freely available tool *MARSCAN* (http://www.bioinformatics.nl/cgi-bin/emboss/marscan) [20].

## 3. Results

**Figure 1:**
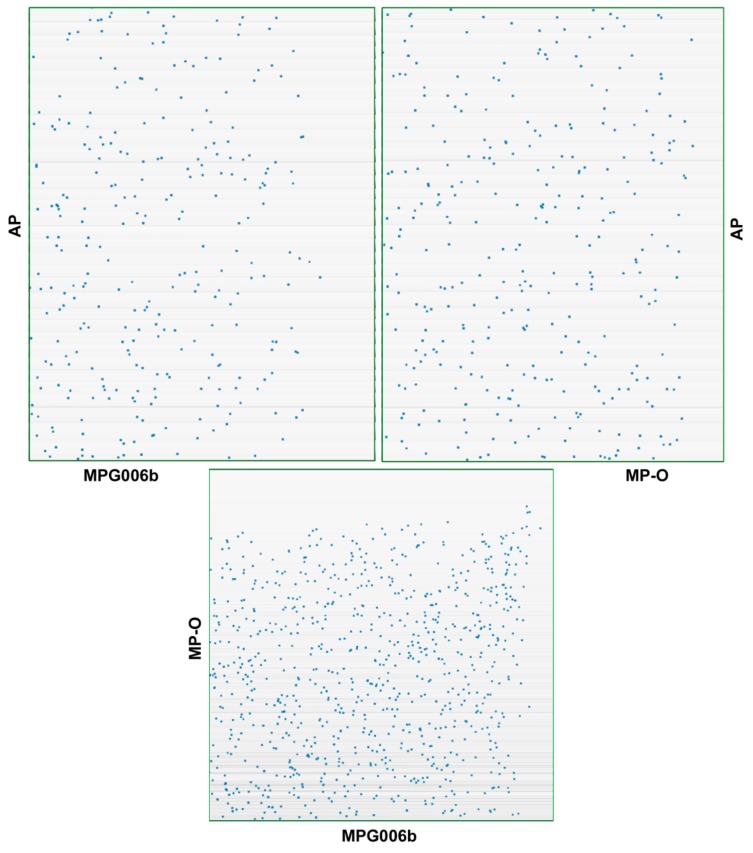
Dot plot comparison (visualized as 2D scatter plots) of *M. persicae* clones O (MP-O) and G006 (MP-G006) reveals a greater similarity between the two *M. persicae* clones in contrast to *A. pisum* (AP).

**Figure 2:**
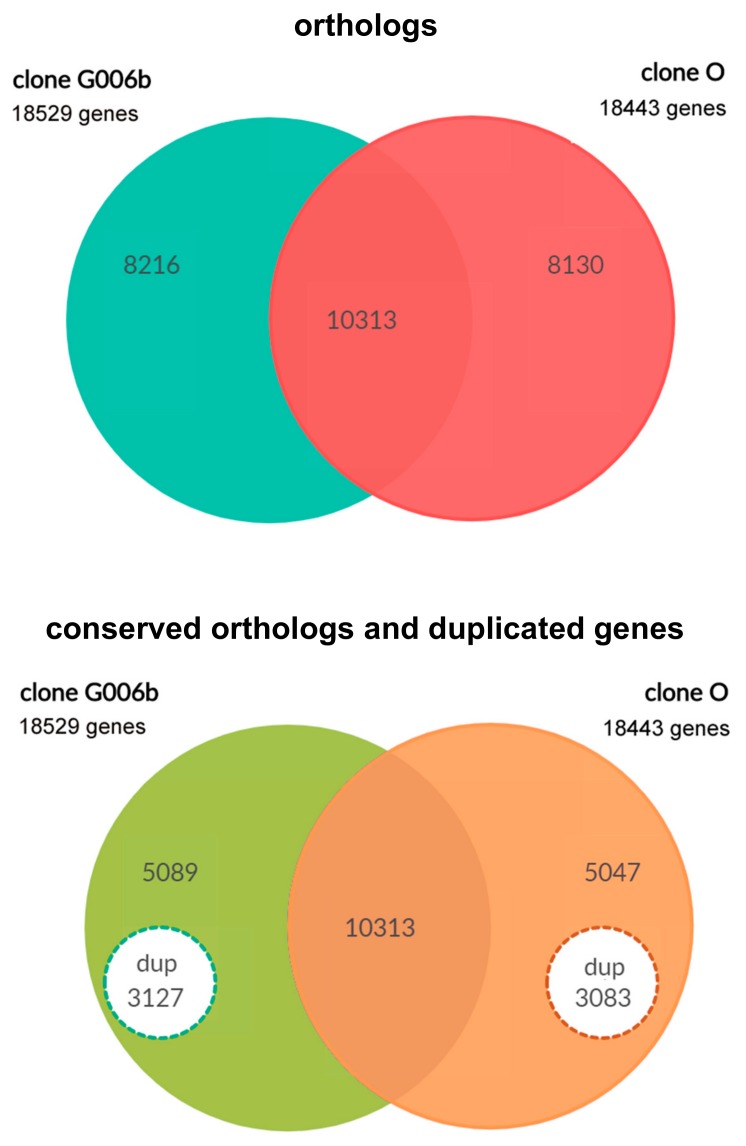
Venn diagram showing the number of orthologues in the two *M. persicae* clones and the number of genes involved in duplications (dup) and those in single copy in each clone.

The combined use of *SynFind* and *SynMap* allowed the identification of the set of orthologous genes in the two *M. persicae* clones (Table 2) and their comparison (Figure 1 and Figure 2, Table 3). This approach showed that about half of the identified genes (55.9%) had an orthologue in the two *M. persicae* clones (depth 1), 27.4% of the annotated genes in clone G006 genes were deleted/rearranged in clone O (depth 0), whilst 15.4% were duplicated (depth 2) or present in more than two copies, since 1.3% of genes were duplicated twice (depth 3) and 0.05% three times (depth 3) in clone G006 in comparison with clone O. Clone O showed 30.5% of genes involved in clone-specific deletions (depth 0), 57.2% had an orthologue in the two *M. persicae* clones (depth 1), and 4.3% of genes were duplicated (depth 2).

The analysis of *intra*- and *inter*-specific genome variation identified 2628 orthologous genes conserved among *A. pisum* and the two *M. persicae* clones, whereas 363 genes were conserved in *A. pisum* and *M. persicae* clone G006b (but not in *M. persicae* clone O); 140 genes were conserved in *A. pisum* and *M. persicae* clone O, but absent in *M. persicae* clone G006b (Supplementary Tables S1 and S2).

**Figure 3:**
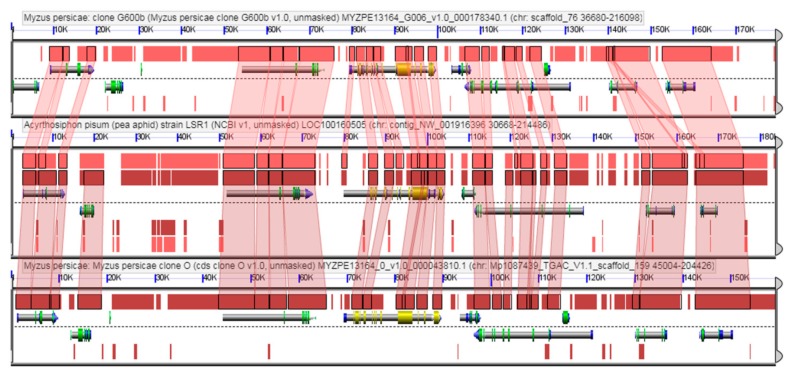
Example of synteny analysis performed comparing the genomes of *M. persicae* clone G006b (top), *A. pisum* (centre) and *M. persicae* clone O (bottom). Genes are displayed as grey bars. Red connectors evidence orthologous regions/genes among species/biotypes and their position in the displayed contig sequence. The complete list of the syntenic regions identified in the *M. persicae* clones is present in Supplementary Table S2 (including the link to each synteny map).

The analysis of syntenic regions evidenced an average of 3.7 genes per syntenic block in the comparison between the two *M. persicae* clones (Figure 3), whereas an average of 2.8 and 2.9 genes per syntenic block was found on comparison of *A. pisum* with *M. persicae* clones O and G006, respectively.

**Figure 4:**
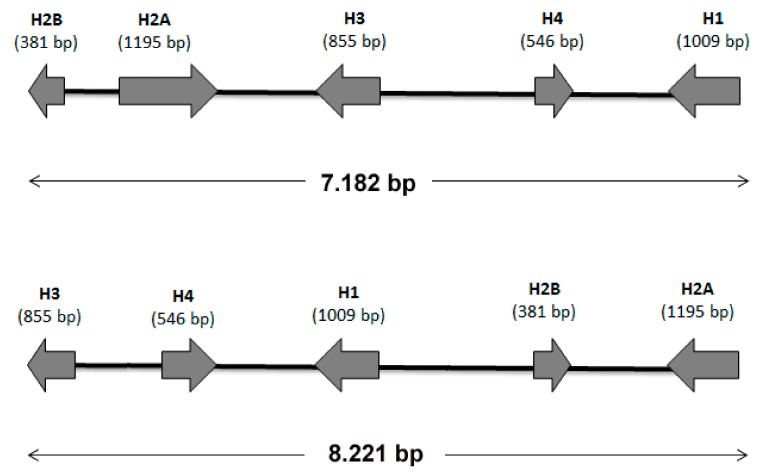
Schematic representation of histone clusters identified in contigs 294 (top) and 1711 (bottom). The length (in bp) of the coding sequence is reported for each histone gene.

In order to identify a molecular marker that could help in the evaluation of the genome quality (in addition to classical features, such as N50 and contig length/number), we evaluated the structure of the gene clusters coding for the five histone proteins H1, H2A, H2B, H3 and H4. Using BLAST analysis no complete histone gene cluster was observed in clone O, whereas the contigs 294 and 1711 of the *M. persicae* clone G006 contained a complete histone gene cluster (Figure 4). However, the two identified histone clusters were different both in length and gene order (Figure 4).

**Figure 5:**
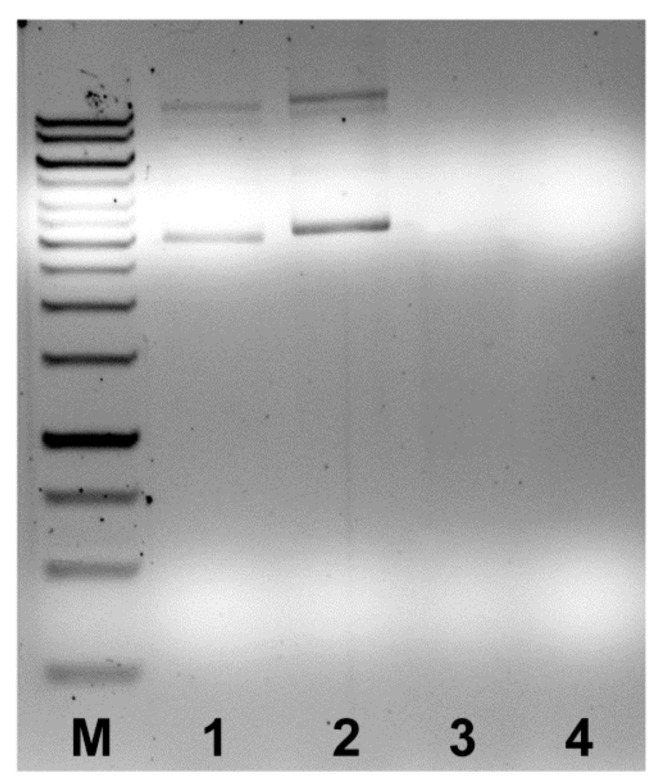
Agarose gel electrophoresis showing the results of the histone cluster PCR amplification with the primers designed on contigs 294 (lanes 1 and 2) and 1711 (lanes 3 and 4) in *M. persicae* clone O (lanes 1 and 3) and G006b (lanes 2 and 4).

**Figure 6:**
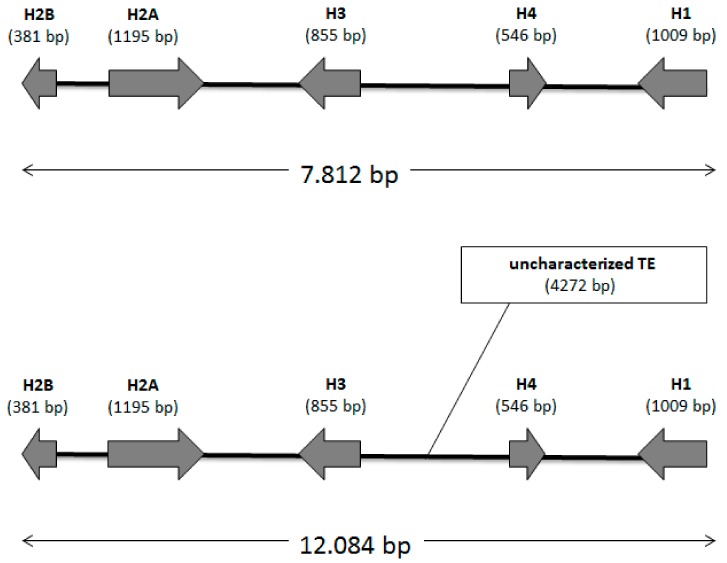
Schematic representation of two histone clusters identified in contig 294 showing the presence of a mobile element in the longer cluster. The length (in bp) of the coding sequence is reported for each histone gene.

To verify whether both the histone clusters were present in *M. persicae* or if they could result from a chimeric assemblage, two specific couples of primers were designed to perform long PCR amplification. This experimental approach revealed that only the histone cluster identified in the contig 294 was present in both the *M. persicae* clones tested (Figure 5). Surprisingly, PCR amplification showed two bands, suggesting that two different histone clusters could be present in each clone, so that the amplified DNA fragments were thus sequenced and aligned. Sequence analysis showed that the two histone clusters have the same sequence, but the longer histone cluster results from the insertion of an uncharacterized transposable element (Figure 6).

**Figure 7:**
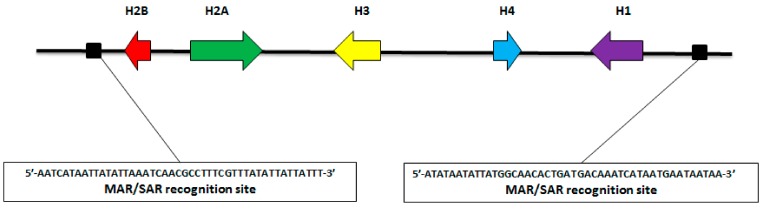
Schematic representation of the *M. persicae* histone cluster together with the two flanking MRS sequences.

**Figure 8:**
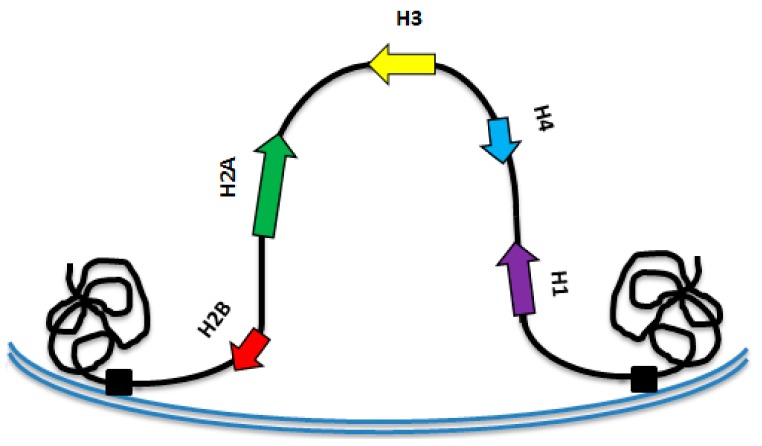
Schematic representation of the putative structural conformation of contigs containing the histone gene cluster in the *M. persicae* nucleus.

Considering that the histone gene cluster is generally flanked by matrix recognition sites (MRS), we performed a MRS search in the *M. persicae* sequences flanking the histone clusters, thereby revealing their presence on both sides (Figure 7). From these data, we infer that a putative folding of the *M. persicae* chromatin occurs in the interphase nuclei so that the histone cluster is located internally in the nucleus with respect to the flanking regions (Figure 8).

## 4. Discussion

The genomes of an increasing number of aphid species have been sequenced in the last decade [8,10,11], but few studies have analysed the differences occurring among biotypes/clones belonging to the same aphid species in order to understand the effects of adaptation to different host plants and clonal selection at a genome scale [22,23,24,25,26].

At present, the genomes of two different clones of *M. persicae* (clones G006 and O) are available [23], together with two genomes belonging to the soybean aphid *A. glycines* [10]. However, the genome of *A. glycines* biotype 1 has been obtained from an aphid population collected on soybean plants in Illinois, USA, whereas the second *A. glycines* genome (generally referred as “hybrid” genome) has been obtained by mixing different natural and laboratory populations collected on soybean plants [10], so that it represents a mix of different *A. glycines* genomes thereby making it much less useful in comparison with other biotypes. 

*M. persicae* clone O was sampled in the UK from Chinese cabbage plants, *Brassica rapa* [26], whereas clone G006 was collected from pepper, *Capsicum, annuum* in Geneva, Switzerland [26] making the comparison of their genomes useful in evaluating the extant of genome conservation in clones collected in very different habitats [18,27]. Furthermore, an *intra*-specific comparison could well improve our knowledge about the genome of each aphid species and validate genomic data, more especially considering that aphid genomes are still scattered in thousands of contigs.

The combined use of *SynFind* and *SynMap* allowed the identification of orthologous genes in the *M. persicae* clones not only in term of their sequence, but also concerning their presence in syntenic regions. Synteny can be extremely useful to confirm gene homology since it makes data more reliable than the identification of orthologues inferred in relation to sequence similarity alone [16]. Furthermore, standard approaches based on clustering algorithms, such as *OrthoMCL* [28] and *INPARANOID* [29], allow successful identification of single copy gene families, but homology cannot be confirmed in the presence of paralogues that are very common in aphids, so that we have included gene positional data in our own analyses.

Synteny analysis clearly showed that in *M. persicae* about 30% of the orthologues were involved in clone-specific deletions/rearrangements. Even considering the high number of contigs that are present in these genomes, these differences are greater than expected in comparison with, for example, *Drosophila melanogaster* populations, thus suggesting that the combination of apomictic parthenogenetic propagation and holocentric chromosomes as found in aphids could favour clone diversification. These results clearly indicate that the choice of performing genome sequencing mixing populations/clones, as done in the case of the soybean aphid *A. glycines* [10], could prove much less useful in attempts to understand aphid molecular ecology, since this approach may well underestimate the difference occurring among biotypes/populations. 

The study of synteny among clones is useful not only to infer homology relationships between genes located within a common genomic neighbourhood [12,30,31,32], but also to better understand the biological specificity of each clone. Indeed, orthologous genes localized at non-syntenic locations generally have relevant differences in their expression pattern, since the different chromosomal localizations may be associated with diverse regulatory sequences [33]. For instance, metabolic responses to insecticides could greatly differ among aphid clones as a consequence of genome rearrangements that in turn could result into diffuse *position*-*effect variegations* [24,25], so that future analyses of the genome of each aphid biotypes could foster the development of more efficient aphid control strategies.

Previous synteny analyses on aphids have been performed comparing species using manually selected sets of genes [16,18]. Until now, the use of manually curated syntenic gene sets is very common and it represents the primary method by which the broader research community has employed such syntenic information in their research. However, manually curated gene sets are generally limited because they generally cover a small portion of the gene repertoire of a species and cannot be updated due to a lag introduced by the time a given genome assembly is published and/or by the existence of updates in data related to genome assemblies, annotations and gene identifiers [16].

Our current analyses gave an average of 3.7 genes per syntenic block in the comparison between the two *M. persicae* clones, but 2.8 and 2.9 genes per syntenic block when the genome of the two *M. persicae* clones were compared to the *A. pisum* genome (in clone O and G006 respectively). Previous studies performed on *A. glycines* [34] revealed on average less than two genes per syntenic block in the comparison with *A. pisum* suggesting that gene order in aphid chromosomes is hardly conserved among species due to the holocentric nature of their chromosomes.

Interestingly, even if these data could be at least partially influenced by the presence of thousands of contigs in the aphid genomes here analysed, the observed synteny strongly mirror data found in Lepidoptera by d' Alençon et al. [35] comparing *Bombyx mori* and the noctuid moth species *Spodoptera frugiperda* and *Helicoverpa armigera.* Indeed, they showed small conserved syntenic blocks of genes approximately containing 1.3 genes per block between *B. mori* and the two noctuid species and 2.0 genes per block between *S. frugiperda* and *H. armigera*. As suggested by d'Alençon et al. [35], this corresponds to approximately two chromosome breaks per Mb of DNA per million years pointing to an evolutionary rate that is much higher than that found among species in the genus *Drosophila* [35]. As a whole, these data support the hypothesis that holocentric chromosomes can favour local chromosomal rearrangements in Lepidoptera and probably in aphids also at the clone/biotype level. 

A further comparison between the two *M. persicae* clones has been performed looking at the structure of the gene cluster coding for the four core histones (H2A, H2B, H3 and H4) and the linker histone (H1). According to the published literature, in insects these are typically clustered in quintets (H2A, H2B, H3, H4 and H1), although scattered solitary histone genes have also been reported [36,37,38]. 

Both the *M. persicae* clones presented a histone gene cluster consisting of the five histone coding genes in the order H2B-H2A-H3-H4-H1 with the cluster spanning more than 7kb. This size is similar to that observed in the pea aphid *A. pisum* [39], confirming that aphids possess larger histone clusters compared with other insects, such as different *Drosophila* species [36].

Furthermore, even if both the aphid species here compared in terms of their genomes presented a typical insect histone gene cluster, where H2A and H2B are adjacent and transcribed in opposite directions and genes H3 and H4 constitute an oppositely transcribed pair [40], the gene order observed in *M. persicae* is different from that reported in *A. pisum,* whereupon the order did not vary in more than 20 *Drosophila* species studied [41]. This reinforces the view that aphid species (and probably also parthenogenetic aphid clones/biotypes) possess a number of genomic rearrangements greater than that present in many other insects.

## 5. Conclusions

This study suggests that *M. persicae* clones are more different at the genome level than previously expected and that the holocentric nature of the aphid chromosomes, together with the presence of apomictic parthenogenesis, can greatly favour chromosomal rearrangements and their inheritance. Even if two clones are not sufficiently numerous to study the effect of *M. persicae* adaptation to different plant hosts, data as here reported prompts further genomic studies aimed at the comparison of such aphid populations adapted to different hosts, especially crops. These data could also help to clarify the real occurrence of generalism and specialisms in aphid feeding [42].

## Figures and Tables

**Table 1:** Sequence and melting temperature of the primers designed to amplify the histone cluster identified in the contigs 294 and 1711. For both the primer couples an annealing temperature of 54.8 °C was used with a polymerase extension at 67 °C for 15 minutes (25 cycles), according to the manufacturer’s instructions.

Primer	T_m_ (°C)	Sequence (5′- 3′)
294_clu-hist_F	59.93	ATTTGGAGCTGGTGTACTTGGT
294_clu-histR	60.52	TGCAATGCATTATCACAAACG
1711_clu-hist_F	60.30	TCGAACAGACCGACCAACTAG
1711_clu-hist_R	59.83	TGCAAATGTCTGGGAATGATAC

**Table 2:** A complete gene list for each *M. persicae* clone has been compiled and data are available at the reported addresses in the *COGE* web server [21].

Clone	Address
*M. persicae* clone G006	https://genomevolution.org/r/15r3b
*M. persicae* clone O	https://genomevolution.org/r/15r40

**Table 3:** Synteny evaluated in the comparison between the *M. persicae* clones G006 and O. Synteny has been classified as “syntenic depth” according to the *SynFind* output.

Syntenic Depth	G006 vs O	O vs G006
0	27.37%	38.50%
1	55.86%	57.19%
2	15.38%	4.32%
3	1.34%	0
4	0.05%	0

## Supplementary Materials

The following are available online at https://www.mdpi.com/2075-4450/10/10/368/s1, Table S1: orthologous mastergene list in *A. pisum* and *M. persicae* clones G006 and O. sUPPL: orthologous mastergene list in *M. persicae* clones G006 and O.

## References

[1] Honeybee Genome Sequencing Consortium (2006). Insights into social insects from the genome of the honey bee. Nature.

[2] Grimmelikhuijzen C.J., Cazzamali G., Williamson M., Hauser F. (2007). The promise of insect genomics. Pestic. Manag. Sci..

[3] Tribolium Genome Sequencing Consortium (2008). The genome of the model beetle and pest *Tribolium castaneum*. Nature.

[4] Arensburger P., Megy K., Waterhouse R.M., Abrudan J., Amedeo P., Antelo B., Bartholomay L., Bidwell S., Caler E., Camara F. (2010). Sequencing of *Culex quinquefasciatus* establishes a platform for mosquito comparative genomics. Science.

[5] Chilana P., Sharma A., Rai A. (2012). Insect genomic resources: Status, availability and future. Curr. Sci..

[6] Mesquita R.D., Vionette-Amaral R.J., Lowenberger C., Rivera-Pomar R., Monteiro F.A., Minx P., Spieth J., Carvalho A.B., Panzera F., Lawson D. (2015). Genome of *Rhodnius prolixus*, an insect vector of Chagas disease, reveals unique adaptations to hematophagy and parasite infection. Proc. Natl. Acad. Sci. USA.

[7] Dudchenko O., Batra S.S., Omer A.D., Nyquist S.K., Hoeger M., Durand N.C., Shamim M.S., Machol I., Lander E.S., Aiden A.P. (2017). *De novo* assembly of the *Aedes aegypti* genome using Hi-C yields chromosome-length scaffolds. Science.

[8] International Aphid Genomics Consortium (2010). Genome sequence of the pea aphid *Acyrthosiphon pisum*. PLoS Biol..

[9] Nicholson S.J., Nickerson M.L., Dean M., Song Y., Hoyt P.R., Rhee H., Kim C., Puterka G.J. (2015). The genome of *Diuraphis noxia*, a global aphid pest of small grains. BMC Genom..

[10] Wenger J.A., Cassone B.J., Legeai F., Johnston J.S., Bansal R., Yates A.D., Coates B.S., Pavinato V.A., Michel A. (2017). Whole genome sequence of the soybean aphid, *Aphis glycines*. Insect Biochem. Mol. Biol..

[11] Quan Q., Hu X., Pan B., Zeng B., Wu N., Fang G., Cao Y., Chen X., Huang Y., Zhan S. (2019). Draft genome of the cotton aphid *Aphis gossypii*. Insect Biochem. Mol. Biol..

[12] Tagu D., Dugravot S., Outreman Y., Rispe C., Simon J.C., Colella S. (2010). The anatomy of an aphid genome: From sequence to biology. C. R. Biol..

[13] Jaquiéry J., Peccoud J., Ouisse T., Legeai F., Prunier-Leterme N., Gouin A., Nouhaud P., Brisson J.A., Bickel R., Purandare S. (2018). Disentangling the causes for faster-X evolution in aphids. Genome Biol. Evol..

[14] Salzberg S.L., Church D., Di Cuccio M., Yaschenko E., Ostell J. (2004). The genome assembly archive: A new public resource. PLoS Biol..

[15] Muggli M.D., Puglisi S.J., Ronen R., Boucher C. (2015). Misassembly detection using paired-end sequence reads and optical mapping data. Bioinformatics.

[16] Tang H., Bomhoff M.D., Briones E., Zhang L., Schnablw J.C., Lyon E. (2015). *SynFind*: Compiling syntenic regions across any set of genomes on demand. Genome Biol. Evol..

[17] Lyons E., Pedersen B., Kane J., Freeling M. (2008). The value of non-model genomes and an example using *SynMap* within CoGe to dissect the hexaploidy that predates rosids. Trop. Plant Biol..

[18] Mandrioli M., Zambonini G., Manicardi G.C. (2017). Comparative gene mapping as a tool to understand the evolution of pest crop insect chromosomes. Int. J. Mol. Sci..

[19] Untergasser A., Cutcutache I., Koressaar T., Ye J., Faircloth B.C., Remm M., Rozen S.G. (2012). Primer3: new capabilities and interfaces. Nucleic Acids Res..

[20] Liebich I., Bode J., Reuter I., Wingender E. (2002). Evaluation of sequence motifs found in scaffold/matrix-attached regions (S/MARs). Nucleic Acids Res..

[21] Lyons E., Freeling M. (2008). How to usefully compare homologous plant genes and chromosomes as DNA sequences. Plant J..

[22] Mandrioli M., Borsatti F. (2007). Analysis of heterochromatic epigenetic markers in the holocentric chromosomes of the aphid *Acyrthosiphon pisum*. Chromosome Res..

[23] Brisson J.A., Davis G.K., Hunter W., Kole C. (2008). Pea Aphid. Genome Mapping and Genomics in Arthropods.

[24] Manicardi G.C., Mandrioli M., Blackman R.L. (2015). The cytogenetic architecture of the aphid genome. Biol. Rev..

[25] Manicardi G.C., Nardelli A., Mandrioli M. (2015). Fast chromosomal evolution and karyotype instability: Occurrence of recurrent chromosomal rearrangements in the peach potato aphid *Myzus persicae* (Hemiptera, Aphididae). Biol. J. Linn. Soc..

[26] Mathers T.C., Chen Y., Kaithakottil G., Legeai F., Mugford S.T., Baa-Puyoulet P., Bretaudeau A., Clavijo B., Colella S., Collin O. (2017). Rapid transcriptional plasticity of duplicated gene clusters enables a clonally reproducing aphid to colonise diverse plant species. Genome Biol..

[27] Monti V., Giusti M., Bizzaro D., Manicardi G.C., Mandrioli M. (2011). Presence of a functional (TTAGG)_n_ telomere-telomerase system in aphids. Chromosome Res..

[28] Li L., Stoeckert C.J., Roos D.S. (2003). OrthoMCL: Identification of ortholog groups for eukaryotic genomes. Genome Res..

[29] Ostlund G., Schmitt T., Forslund K., Köstler T., Messina D.N., Roopra S., Frings O., Sonnhammer E.L. (2010). *InParanoid 7*: New algorithms and tools for eukaryotic orthology analysis. Nucleic Acids Res..

[30] Moreno-Hagelsieb G., Trevino V., Perez-Rueda E., Smith T.F., Collado-Vides J. (2001). Transcription unit conservation in the three domains of life: A perspective from *Escherichia coli*. Trends Genet..

[31] Poyatos J.F., Hurst L.D. (2007). The determinants of gene order conservation in yeasts. Genome Biol..

[32] Heger A., Ponting C.P. (2007). Evolutionary rate analyses of orthologs and paralogs from 12 *Drosophila* genomes. Genome Res..

[33] Davidson R.M., Gowda M., Moghe G., Lin H., Vaillancourt B., Shiu S.H., Jiang N., Robin Buell C. (2012). Comparative transcriptomics of three Poaceae species reveals patterns of gene expression evolution. Plant J..

[34] Mandrioli M., Melchiori G., Panini M., Chiesa O., Giordano R., Mazzoni E., Manicardi G.C. (2019). Analysis of the extent of synteny and conservation in the gene order in aphids: A first glimpse from the *Aphis glycines* genome. Insect Biochem. Mol. Biol..

[35] d’Alençon E., Sezutsu H., Legeai F., Permal E., Bernard-Samain S., Gimenez S., Gagneur C., Cousserans F., Shimomura M., Brun-Barale A. (2010). Extensive synteny conservation of holocentric chromosomes in Lepidoptera despite high rates of local genome rearrangements. Proc. Natl. Acad. Sci. USA.

[36] Lifton R.P., Goldberg M.L., Karp R.W., Hogness D.S. (1977). The organization of the histone genes in *Drosophila melanogaster*: Functional and evolutionary implications. Cold Spring Harb. Symp. Quant. Biol..

[37] Engel J.D., Dodgson J.B. (1981). Histone genes are clustered, but not tandemly repeated in the chicken genome. Proc. Nat. Acad. Sci. USA.

[38] Maxon R., Cohn R., Kedes L. (1983). Expression and organization of histone genes. Annu. Rev. Genet.

[39] Mandrioli M., Manicardi G.C. (2013). Chromosomal mapping reveals a dynamic organization of the histone genes in aphids (Hemiptera: Aphididae). Entomologia.

[40] Roehrdanz R., Heilmann L., Senechal P., Sears S., Evenson P. (2010). Histone and ribosomal RNA repetitive gene clusters of the boll weevil are linked in a tandem array. Insect Mol. Biol..

[41] Strausbaugh L.D.M., Weinberg E.S. (1982). Polymorphism and stability in the histone gene cluster of *Drosophila melanogaster*. Chromosoma.

[42] Loxdale H.D., Balog A., Harvey J.A. (2019). Generalism in Nature…The great misnomer: Aphids and wasp parasitoids as examples. Insects.

